# A Transposon-Derived DNA Polymerase from *Entamoeba histolytica* Displays Intrinsic Strand Displacement, Processivity and Lesion Bypass

**DOI:** 10.1371/journal.pone.0049964

**Published:** 2012-11-30

**Authors:** Guillermo Pastor-Palacios, Varinia López-Ramírez, Cesar S. Cardona-Felix, Luis G. Brieba

**Affiliations:** 1 Laboratorio Nacional de Genómica para la Biodiversidad, Centro de Investigación y de Estudios Avanzados del Instituto Politécnico Nacional, Irapuato, Guanajuato, México; 2 Departamento de Ingeniería Genética, Centro de Investigación y de Estudios Avanzados del Instituto Politécnico Nacional, Centro de Investigación y de Estudios Avanzados del Instituto Politécnico Nacional, Irapuato, Guanajuato, México; Institute of Enzymology of the Hungarian Academy of Science, Hungary

## Abstract

*Entamoeba histolytica* encodes four family B2 DNA polymerases that vary in amino acid length from 813 to 1279. These DNA polymerases contain a N-terminal domain with no homology to other proteins and a C-terminal domain with high amino acid identity to archetypical family B2 DNA polymerases. A phylogenetic analysis indicates that these family B2 DNA polymerases are grouped with DNA polymerases from transposable elements dubbed Polintons or Mavericks. In this work, we report the cloning and biochemical characterization of the smallest family B2 DNA polymerase from *E. histolytica*. To facilitate its characterization we subcloned its 660 amino acids C-terminal region that comprises the complete exonuclease and DNA polymerization domains, dubbed throughout this work as EhDNApolB2. We found that EhDNApolB2 displays remarkable strand displacement, processivity and efficiently bypasses the DNA lesions: 8-oxo guanosine and abasic site.

Family B2 DNA polymerases from *T. vaginalis*, *G. lambia* and *E. histolytica* contain a Terminal Region Protein 2 (TPR2) motif twice the length of the TPR2 from φ29 DNA polymerase. Deletion studies demonstrate that as in φ29 DNA polymerase, the TPR2 motif of EhDNApolB2 is solely responsible of strand displacement and processivity. Interestingly the TPR2 of EhDNApolB2 is also responsible for efficient abasic site bypass. These data suggests that the 21 extra amino acids of the TPR2 motif may shape the active site of EhDNApolB2 to efficiently incorporate and extended opposite an abasic site. Herein we demonstrate that an open reading frame derived from Politons-Mavericks in parasitic protozoa encode a functional enzyme and our findings support the notion that the introduction of novel motifs in DNA polymerases can confer specialized properties to a conserved scaffold.

## Introduction

The genome of *Entamoeba histolytica* contains replicative DNA polymerases α, δ and ε; lesion repair polymerases Rev1 and Rev3, and a family A DNA polymerase able of thymine glycol bypass [1], [2], [3]. Protozoan parasites *Trichomonas vaginalis, Giardia lambia* and *E. histolytica* encode a great variety of transposable elements (TEs) [4]. Among these TEs, a novel class of DNA transposons dubbed Polintons or Mavericks are elements of 15 to 20 kb that encode a family B2 DNA polymerase, a retroviral integrase, a protease and a putative ATPase [5], [6]. It is suggested that Politons-Mavericks maybe related to double-stranded DNA viruses and have a direct influence in the evolution of these parasites [7]. For instance, it is estimated that 5% of the genome of *T. vaginalis* consists of multiple copies of Polintons-Mavericks [5], [6]. DNA polymerases from Polinton-Mavericks have to efficiently replicate these long repetitive DNA elements. However, to date no studies on the biochemical characterization of proteins involved in the replication process of Politons-Mavericks have been carried out in any organism. In principle, family B2 DNA polymerases from Politons-Mavericks must be highly proccesive in order to be able to replicative over 20 kbs [5], [6], [7]. Family B2 DNA polymerases are modular proteins that contain a polymerization and a 3′–5′ exonuclease domain and two extra elements dubbed Terminal Protein Regions (TPR) 1 and 2. The polymerization is divided in 3 subdomains: thumb, fingers and palm. The structural arrangement of these subdomains forms a cupped right hand in which a double stranded DNA is positioned for nucleotide addition [8], [9].

Nature has found two structural solutions for DNA polymerases to incorporate thousands of nucleotides without falling off a template strand. One is the use of processivity factors, like torodial shape proteins or factors that encircle or increment the surface/area between a DNA polymerase and double stranded DNA substrate, such as PCNA, β-clamp, thioredoxin, UL44, and the β subunit of DNA polymerase γ [10], [11], [12], [13]. The second solution is to confer intrinsic processivity to replicative DNA polymerases by the addition of novel domains, as it occurs in T5 and φ29 DNA polymerases [14], [15]. φ29 DNA polymerase is the archetypical family B2 DNA polymerase and its TPR2 is responsible for processivity and strand displacement [16], [17]. TPR2 structurally resembles the promoter specificity loop of single subunit RNA polymerases, suggesting that nature has used the two beta strand extended loop for processivity and promoter selectivity and that the presence of this loop may have occurred before the specialization of single subunit DNA and RNA polymerases [18], [19]. Family B2 DNA polymerases are present in bacteriophages, yeast, cnidarians and parasitic protozoa [20]. However, the only family B2 DNA polymerases characterized to date are those from phages.

A recent report corroborates that *E. histolytica* contains four family B2 DNA polymerases [21], however the report only characterized its cellular localization and *in vivo* expression. Herein we report the biochemical characterization of a family B2 DNA polymerase from *E. histolytica*, this polymerase in comparison to φ29 DNA polymerase contains 21 amino acids extra in its TPR2. We found that this extended TPR2 is responsible for processive polymerization, strand displacement and abasic site lesion bypass.

## Materials and Methods

### Phylogenetic analysis and structural modeling of EhDNApolB2

Putative family B2 DNA polymerase were searched in Pathema database (http://pathema.jcvi.org/Pathema/) using the amino acid sequence of φ29 DNA polymerase (**Table S1**). Initial amino acid alignment was carried out with ClustalW and manually corrected. Phylogenetic reconstruction of the family B2 DNA polymerase sequences was obtained using the maximum likelihood method with LG+I+G as substitution model with gamma = 1.66 and 1,000 bootstrap replicates on phyML 3.0 program at the web server (http://www.lirmm.fr/~gascuel) [22] (**Table S2**). The homology model of EhDNApolB2 was constructed using the Molecular Operating Environment (MOE) program with the crystal structure of the complex φ29 DNA polymerase/primer-template DNA as template (PDB ID: 2PZS) [19].

### Cloning, Protein expression and purification

**Figure 1:**
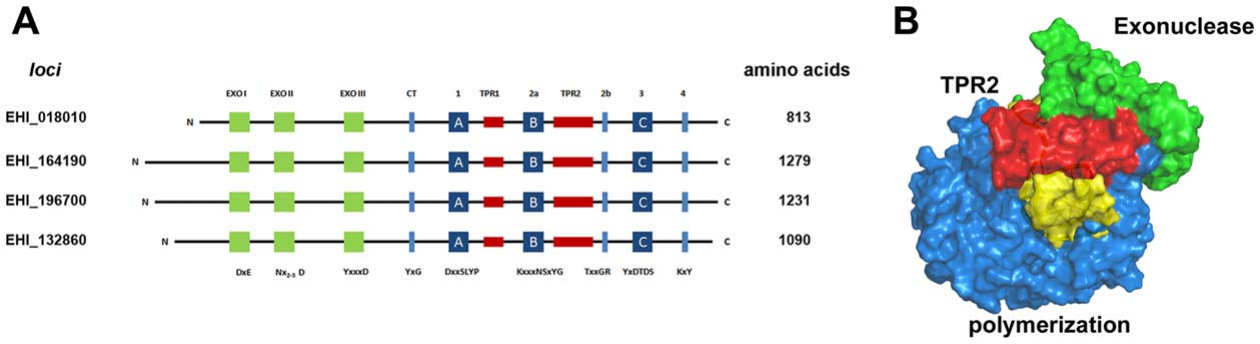
Modular organization of family B2 DNA polymerases in *E. histolytica* and structural model of EhDNApolB2. (**A**) *E. histolytica* contains four family B2 DNA polymerases in its genome. These DNA polymerase present a C-terminal region with conserved exonuclease and polymerase motifs characteristic of a family B2 DNA polymerases (green, blue and red boxes). The N-terminal region, indicated by a thin line, presents no similitude to other proteins and is composed of 180 to 500 amino acids. The shortest DNA polymerase is present at loci EHI_018010 and is dubbed EhDNApolB2 throughout this work (**B**) Homology structural model of EhDNApolB2. The 3′–5′ exonuclease domain is shown in green and the 5′–3′ polymerization domain is shown in blue. The extended TPR2 motif is shown in red encircling double stranded DNA (yellow colored).

Full-length ORF located in loci EHI_018010 and a N-terminal 153 amino acids deletion were PCR amplified using oligonucleotides directed from the Pathema database (**Table S3**) and subcloned into the pCOLD I vector (Takara). As full-length EHI_018010 is poorly expressed in *E. coli*, through this work we focused on the N-terminal deletion that we dubbed EhDNApolB2 (**Figure 1**). The pCOLDI-EhDNApolB2 construct was transformed into *E. coli* BL21 DE3-Rosseta II. Transformants were inoculated into 50 ml of LB supplemented with 100 µg/ml of ampicillin and 35 µg/ml of chloramphenicol and used to inoculate 1 liter of LB. This culture was grown at 37°C until it reached an OD600 of 0.6 and induced with 0.5 mM IPTG for 16 hours at 16°C. The cell pellet was harvested by centrifugation at 4°C. Bacterial lysis was carried out by the freeze-thawing method; briefly the pellet was resuspended in 40 ml of 50 mM potassium phosphate pH 8, 300 mM NaCl, 1 mM PMSF, 0.5 mM DTT and 0.5 mg/ml of lysozyme, incubated on ice for 30 minutes and freeze-thaw two times. The resuspended cell culture was sonicated and centrifuged at 17,000 rpm for 30 minutes at 4°C. Recombinant EhDNApolB2 was purified by Immobilized Metal Affinity Chromatography (IMAC) using a 1 ml pre-packed column. The eluate was dialyzed in 50 mM potassium phosphate pH 7.0, 1 mM DTT, 100 mM NaCl and 2 mM EDTA. To further purify EhDNApolB2, the dialyzed protein was loaded onto a heparin column and eluted with a NaCl gradient (50 to 1000 mM). EhDNApolB2 eluted between 400 to 700 mM of NaCl. The fractions were dialyzed in 50 mM potassium phosphate pH 7.0, 1 mM DTT, 100 mM NaCl, 0.5 mM EDTA, 50% glycerol and stored at −20°C. Purity was verified on a 10% SDS-PAGE stained with Coomassie Brilliant Blue R-250.

### Deletion and site directed mutagenesis

Exonuclease deficient EhDNApolB2 was constructed by mutating residue Asp345 to alanine using the Quick-Change protocol (Stratagene) accordingly to the manufacturer instructions. ΔTPR2 DNA polymerase mutant was obtained by deletional PCR mutagenesis using Phusion DNA polymerase (Finnzyme) with primers designed to flanking the ends of the TPR2 region. The complete oligonucleotide sequences used is described in **Table S3**. Exonuclease deficient and deletion polymerases were confirmed by automated DNA sequencing.

### Polymerization and degradation reactions

Oligonucleotides were used to generate double stranded polymerization substrates as previously described. For a complete list of oligonucleotides used as substrates please refer to **Table S4**. The nucleotide sequence of the 45 mer template strand is 5′-cct tgg cac tag cgc agg gcc agt tag gtg ggc agg tgg gct gcg-3′ and 24 mer primer sequence is: 5′-cgc agc cca cct gcc cac cta act-3′ [3]. Several rounds of buffer optimization revealed that the optimal primer extension buffer for EhDNApolB2 consists of 50 mM Tris-HCl pH 7.5, 2.5 mM MgCl2, 1.5 mM DTT, 0.2 µg/ml BSA (data not shown). Polymerization reactions were carried out using a final concentration of 1 nM primer-template and 20 nM EhDNApolB2 at 37°C. Proofreading exonuclease activity was performed using a set of double stranded matched and mismatched substrates as indicated in the figure legend. The reactions were carried out to 25°C and stopped to indicate time with stop buffer. Single stranded exonuclease activity was performed by a time course experiment using a 5′ radiolabeled single stranded DNA (**Table S4**) at 37°C. Reactions were stopped with a buffer containing 95% formamide, 50 mM EDTA, 0.01% bromophenol blue.

Reaction mixtures were run on a 15% polyacrylamide gel and 8 M urea. Denaturing polyacrylamide gels were dried and analyzed by phosphorimagery on a Molecular Dynamics PhosphorImager.

### Translesion synthesis

DNA amplifications were carried out using 1 nM DNA and variable polymerase concentration (20 nM EhDNApolB2 or 40 nM ΔTPR2) and 100 µM each dNTP and aliquots were removed as a function of time, added to a stop buffer. Subsequently, samples were run on a 15% polyacrylamide 8 M urea gel and analyzed using a PhosphorImager and Quantity-One software.

### Strand-displacement

Strand displacement was carried out with a template of 45 oligonucleotides and a γ–P^32^ ATP labeled 21mer primer. A third oligonucleotide of 24, 21 and 18 nucleotides was hybridized to create gaps of 1, 3 and 6 nt. Reactions were carried out with 1 nM primer-template, 20 nM of EhDNApolB2 and 40 nM of ΔTPR2 at 37°C. Reaction mixtures were run on a 15% polyacrylamide gel, 8 M urea.

### Processivity Assay

Processivity assays were carried out using single stranded M13mp18 hybridized with a forward primer of 17 nucleotides labeled with γ–P^32^ ATP. The template was present at 1 nM at the polymerases at 20 y 40 nM. Aliquots were taken at 10, 20 and 40 minutes and stopped with equal amounts of 90% formamide, 50 mM EDTA, 0.1% bromophenol blue. Samples were run on a 6% polyacrylamide gel 8 M urea. The dried gel was visualized and analyzed using a PhosphorImager and Quantity-One software.

## Results and Discussion

### Identification of Polinton-Maverick derived family B2 DNA polymerases in *E. histolytica*

We performed a BLAST search in the genome of *E. histolytica* and found 4 family B2 DNA polymerases, although with different amino acid lengths that a previous report which classified them as organellar DNA polymerases [21] (**Figure 1A**). The BLAST search indicates that the closest orthologs to the family B2 DNA polymerases of *E. histolytica* are DNA polymerases related to a TE dubbed Polinton-Maverick present in *Entamoeba invadens*. Our phylogenetic analysis corroborates an initial observation that the four family B2 DNA polymerase of *E. histolytica* are closely related to Polinton-Maverick DNA polymerases from *G. lambia* and *T. vaginalis* [1], [4], [6] (**Figure S1A**). In this analysis the four family B2 DNA polymerases from *E. histolytica* are grouped into well-defined branches with a bootstrap value of 1000 for the nearest branch. Family B2 DNA polymerases from linear protein-primed replicated plasmids and bacteriophages are located in different branches of this phylogenetic tree. Thus, our phylogenetic tree analysis strongly suggests that the four family B2 DNA polymerases from E. *histolytica* are related to TEs. Furthermore, we were able to identify the conserved exonuclease and polymerization motifs of family B and the TPR1 and TPR2 extensions of family B2 DNA polymerases in those DNA polymerases (**Figure 1A** **and Figure S1B**) [23], [24], [25], [26], [27]. The conservation of the critical amino acids involved in catalysis for the polymerization and exonucleolytic domains indicates that all four family B2 DNA polymerase in *E. histolytica* may display polymerase and exonuclease activities (**Figure 1A**). The non-conserved N-terminal segment of Polinton-Maverick derived family B2 DNA polymerases of *E. histolytica* maybe related to a protein segment that functions as a terminal protein as is observed in family B2 DNA polymerases from protein-primed replicated plasmids (For a recent review [28]). We were unable to find the retroviral-like integrase, adenoviral-like protease and ATPase in the genome of *E. histolytica* associated with Polintons-Mavericks. However, the genome of *E. invadens* contains a 16,504 bp Polinton-Maverick that contains these canonical proteins [6] and the associated family B2 DNA polymerase shares approximately 85% amino acid identity in polymerization domain of family B2 DNA polymerases from *E. histolytica*. It is possible that our failure in finding integrase, adenoviral-like protease and a putative ATPase orthologous in *E*. *histolytica* maybe to an error in the genome assembly or due to gene lost.

As the amino acid length of the four family B2 DNA polymerases of *E. histolytica* varies between 813 to 1239 amino acid and the main divergences are located at the N-terminal of these polymerases, we decided to biochemically characterize the ORF in loci EHI_018010 because of its reduced amino length and similitude to the well characterized φ29 DNA polymerase. This polymerase is dubbed in this work “*full-length EhDNApolB2*” (**Figure S1B**). We cloned and sequenced two independent clones of full-length EhDNApolB2. After sequencing them we corrected the identity of several residues located in the exonuclease domain, among them those present in motif Exo III. An amino acid sequence alignment of full-length EhDNApolB2 in comparison to φ29 DNA polymerase indicates that both proteins share 38% amino acid identity in their exonuclease and polymerization domains and that the main difference appears at the length of the TPR2 motif, which is 21 amino acids longer in EhDNApolB2 (**Figure S2**). A structural model of the 3′–5′ exonuclease and polymerase domains of full-length EhDNApolB2 depicts these 21 extra amino acids as two beta strands adjacent to the finger subdomain. In this structural model the TPR2 motif completely encircles the double stranded DNA (**Figure 1B**).

### EhDNApolB2 is an active DNA polymerase

**Figure 2:**
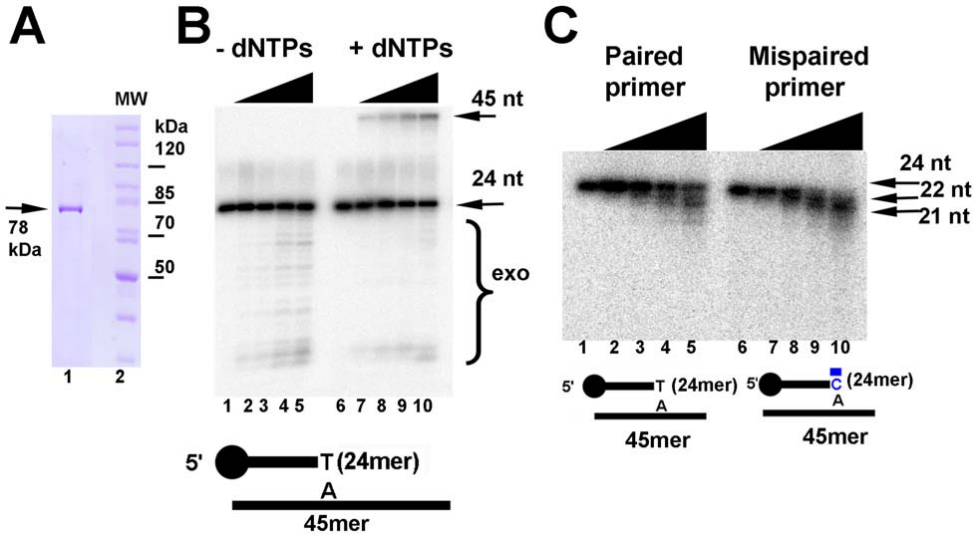
Heterologous purification and enzymatic activities of EhDNApolB2. (**A**) **Purification of EhDNApolB2.** 10% SDS-PAGE Coomassie blue stained showing the final purification of EhDNApolB2 as a single protein band of 78 kDa (lane 1) in relation with molecular weight standards (lane 2). (**B**) **EhDNApolB2 displays 3′–5′ exonuclease and 5′–3′-polymerization activities.** Exonuclease and polymerazation activities were measured using a γ–P^32^ 24mer primer annealed to a complementary 45 nt template at 1 nM. Lanes 1 to 5 contains reactions with out added dNTPs and increasing concentrations of EhDNApolB2 (0, 5, 10, 20 and 30 nM). Reactions in lanes 6 to 10 were incubated with 100 µM of each dNTP. The bottom arrow depicts the primer and the upper arrow depicts the product. Samples were taken at 10 minutes and stopped with 50 mM EDTA and 90% formamide. Incorporation of all dNTP resulted in a band of 45 nt and in the absence of dNTP resulted in processive degradation of the labeled substrate. As observed polymerization and exonuclease activities are in competition (lanes 6 to 10). (**C**) **3′–5′ exonuclease activity on paired and mispaired primer terminus**. 3′–5′ exonuclease time course activity assay with 5′ γ–P^32^ radiolabeled paired (lanes 1 to 5) or mispaired (lanes 6 to 10) primer-templates. Double stranded labeled templates at a final concentration of 1 nM were incubated on ice for 5 minutes with EhDNApolB2 at a final concentration of 20 nM in standard reaction buffer with out divalent metal. Exonucleolytic reaction was initiated by adding MgCl_2_ at a final concentration of 2.5 mM. The samples were incubated at 25°C and aliquots were taken at 0, 1, 2, 4 and 8 minutes and stopped with 50 mM EDTA and 90% formamide. Samples were run onto a 15% denaturing polyacrylamide gel electrophoresis and analyzed by phosphorimagery.

Protein expression of full-length EhDNApolB2 resulted in a poorly expressed heterologous protein with yield of less than 0.1 mg per liter of bacterial culture (data not shown). In order to circumvent this problem we decided to create a construct in which the first 153 amino acids were eliminated. These 153 amino acids have no homology with any know protein in the GenBank. The deleted protein resembles in length to φ29 DNA polymerase, which is the archetypical family B2 DNA polymerase (**Figure S1B**). The deleted protein was cloned in a pCOLD I vector and purified by IMAC and heparin chromatography with typical yields of 2 mg per liter of cell culture. After these two purification steps, the protein is more than 95% pure as assessed in a denaturating SDS-PAGE and present a molecular weight of 78 kDa (**Figure 2A**). We refer to protein as EhDNApolB2 throughout this work. In order to corroborate the putative 3′–5′ exonuclease and polymerization activities of EhDNApolB2 we measured its enzymatic activities for double stranded DNA exonucleolytic degradation and primer-template polymerization. As observed in **Figure 2B**, in the absence of dNTPs EhDNApolB2 gives rise to degradation products that increase in direct relationship with its concentration, indicating that EhDNApolB2 is an active 3′–5′ exonuclease (**Figure 2B** **, lanes 1 to 5**). In the presence of dNTPs, EhDNApolB2 is able to elongate a 21mer primer to a full-length 45mer product in a concentration-dependent fashion (**Figure 2B** **, lanes 6 to 10**). The exonucleolytic products observed in lanes 6 to 10 indicate that, as is observed for other DNA polymerases, the exonucleolytic and polymerization activities of EhDNApolB2 are in competition, and that the presence of dNTPs shifts the reaction towards the polymerization mode. The 3′–5′ exonuclease domain of DNA polymerases like Klenow Fragment, T7 DNA polymerase or φ29 DNA polymerase is responsible of mismatch proofreading an contributes to overall polymerase fidelity [29], [30]. To investigate the role of the 3′–5′ exonuclease of EhDNApolB2 in proofreading we performed a time course experiment using a primer-template substrate with a paired and a mispaired 3′OH at 25°C (**Figure 2C**). As observed in Figure 2B, the reaction product of EhDNApolB2 after an incubation of 8 minutes with the paired substrate results in approximately 50% of the 24mer hydrolyzed to exonucleolytic products, whereas in the mispaired substrate it has been completely hydrolyzed to smaller 22mer and 21mer products (**Figure 2C** **, lanes 5 and 10**). At 25°C the exonuclease product for the paired nucleotide is limited to 21 nt (**Figure 2B** **, lane 1 to 5**) in contrast to the lower migrating product observed in the exonucleolytic degradation at 37°C (**Figure 2B** **, lanes 1 to 5**). The differential in activity accordingly to the temperature correlates with the shuttle between exonuclease and polymerase active sites; at lower temperature the primer translocates to the polymerase active site before it subsequent hydrolysis. The exonucleolytic degradation differential observed for paired and mispaired substrates is in agreement with the idea that the frayed end of the mispaired primer translocates to the exonuclease active site of EhDNApolB2 more effectively than a paired end. This preference for a mispaired versus a paired primer-template indicate that the 3′–5′ exonuclease domain of EhDNApolB2 contributes to the overall fidelity of this polymerase as is the case for other polymerases [31], [32], [33]. These polymerization and proofreading activities of EhDNApolB2 are in agreement to the presence of 4 invariant amino acids containing carboxylic acid groups (Asp163 and Glu 165, Asp221, and Asp345) in the 3′–5′ exonuclease motifs ExoI, ExoII, and ExoIII of the exonuclease domain, and 3 invariant aspartic acids and a tyrosine residue (Asp430, Tyr 582, Asp673 and Asp675) located in motifs A, C, and B of the polymerization domain of EhDNApolB2 with respect to family B2 DNA polymerases (**Figure S2**).

In order to corroborate that EhDNApolB2 belongs to the family B of DNA polymerases and to further ensure that the observed polymerase and exonuclease activities are intrinsic of EhDNApolB2 and not due to a co-purified DNA polymerase from *E. coli* we tested the effect of aphidicolin, a specific inhibitor of family B DNA polymerases, on EhDNApolB2. Aphidicolin inhibits φ29 DNA polymerase, DNA polymerase α and family B DNA polymerases from archaea like *Aeropyrum pernix* or *Pyrococcus furiosus* [34], [35], [36]. For instance, at a concentration of 10 µM dNTPs, the amount of polymerized substrate by φ29 DNA polymerase is reduced by half in the presence of 400 µM of aphidicolin [34]. In **Figure S3** we shown that in the presence of 25 µM dNTPs, the amount of primer extension of M13 single stranded DNA annealed to a 17mer by EhDNApolB2 is reduced by half at a concentration of 242 µM of aphidicolin. Thus, EhDNApolB2 is sensible to aphidicolin inhibition in a similar level than φ29 DNA polymerase. As in other eukaryotes, aphidicolin inhibits the growth and DNA synthesis of *E. histolytica* indicating the presence of family B2 DNA polymerases in this parasite, as *E*. *histolytica* contain canonical family DNA polymerase (α, δ, and ε) involved in nuclear DNA replication [37].

### EhDNApolB2 efficiently bypasses 8-oxo guanosine and abasic site lesion

**Figure 3:**
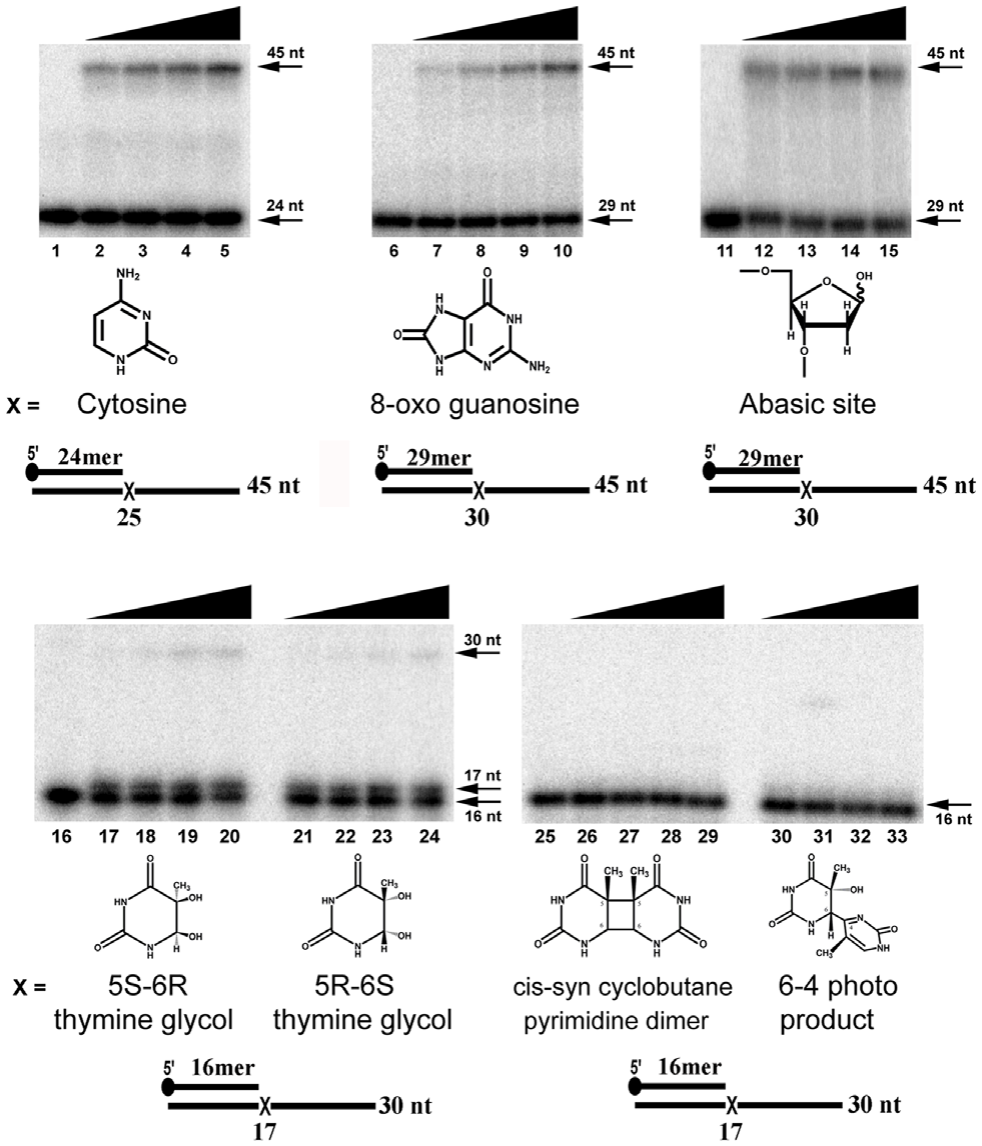
EhDNApolB2 efficiently bypasses 8-oxo guanosine and abasic site lesions. Denaturing polyacrylamide gel electrophoresis showing translesion bypass of EhDNApolB2 in comparison to undamaged template. Primer extension by EhDNApolB2 using a canonical and damaged substrate. The first nucleotide (canonical or damaged) that serves a template is designated by an **X**. For the 8-oxoguanosine and abasic site the lesion is located immediately after a primer of 29 nt and for thymine glycol and UV adducts is located immediately after a primer of 16 nt. The label 25, 30 or 17 nt indicate the length of the primer is only one nucleotide opposite the lesion is incorporated. Each reaction was incubated with a 20 nM of EhDNApolB2 and 1 nM of several substrates. Aliquots were taken at 0, 2.5, 5, 10 and 20 minutes. Time course of different substrates were loaded in a 15% denaturing gel. Thymine (lanes 1–5); 8-oxo guanosine (lanes 6–10); abasic site (lanes 11–15); 5 S-6R thymine glycol (lanes 16–20); 5R-6S thymine glycol (lanes 21–24); cis-syn cyclobutane pyrimidine dimer (lanes 25–29); 6-4 photo product (lanes 30–33); The upper arrow depicts the length of the final product substrate and the bottom arrow indicates the used primer.

*E. histolytica* is exposed to oxidative damage during macrophage attack and genes that cope with free radicals are over-expressed in those conditions [38], [39]. As a nuclear family A DNA polymerase from *E. histolytica* is able to efficiently bypass thymine glycol [3] we determined the lesion bypass of recombinant EhDNApolB2. To determine translesion synthesis of EhDNApolB2, we assayed primer extension using a set of primer-templates in which a specific DNA lesion is to be used as a template immediately after the end of the primer. Thus, the first nucleotide to be incorporated into the primer is incorporated opposite the lesion. In a primer extension experiment in which the first base to be used as a template is a control cytosine, a time course experiment shows that at the shortest time (2.5 min) only 4% of the substrate has been used and at the longest time (20 minutes) 22% of the product is fully extended (**Figure 3** **, lanes 1 to 5**). Lesion bypass was studied using 8-oxoguanosine, abasic site, 5R, 6S and 5S, 6R thymine glycol, cyclobutane prymidine dimer (CPD) and 6-4 product (6-4 PP) (**Figure 3** **, lanes 6 to 33**). We found that EhDNApolB2 efficiently bypasses 8-oxoguanosine and abasic sites, but is unable to bypass thymine glycol, cyclobutane prymidine dimer and 6-4 product (**Figure 3**). EhDNApolB2 efficiently incorporates and extends from 8-oxoguanosine, after an incubation of 20 minutes 20% of the primer-template is utilized (**Figure 3** **, lanes 6 to 10**). 8-oxoguanosine only posses a moderate block to replicate DNA polymerase and it is readily bypassed by orthologous DNA polymerases like φ29 DNA polymerase [40], [41]. Interestingly primer extension of an abasic site containing template is of 12% at the shortest incubation time and 25% complete at the longer extension time, indicating that in this experiment the abasic site is utilized with similar efficiency that an undamaged base (**Figure 3** **, lanes 11 to 15**). The only other known DNA polymerase able to incorporate and extend opposite an abasic site with high efficiency is DNA polymerase θ [42]. Y-family DNA polymerases can incorporate opposite an abasic site but are only moderately active to extend after nucleotide incorporation [43].

Thymine glycol posses a strong block for family B polymerase that only incorporate one nucleotide opposite to this lesion and are unable of bypass. EhDNApolB2 bypasses 5R, 6S thymine glycol and 5S, 6R thymine glycol lesions with very low efficiency. The amount of full-length product after 20 minutes is 2 and 3% for 5R, 6S thymine glycol and 5S, 6R thymine glycol respectively. As in other DNA polymerases EhDNApolB2 incorporates only one nucleotide in 5R, 6S thymine glycol and 5S, 6R thymine glycol substrates [44]. The percentage of single nucleotide addition is 38 and 43% respectively (**Figure 3** **lanes 16 to 24**). However, EhDNApolB2 only incorporates one nucleotide opposite thymine glycol, and is not able to extend after single nucleotide incorporation. This is in contrast to DNA polymerase ν and a family A DNA polymerase from *E. histolytica* which efficiently bypass thymine glycol [3], [42]. RB69 DNA polymerase incorporates one nucleotide opposite thymine glycol, but it does not elongate from it indicating that family B DNA polymerase are unable to bypass thymine glycol [45]. Structural studies indicate that the extra methyl group of thymine glycol displaces the incoming template base into a non catalytically competent conformation for further elongation [45]. The low percentage of thymine glycol by EhDNApolB2 may indicate a more flexible active site in comparison to other family B DNA polymerases. As expected EhDNApolB2 is unable to bypass CPD and 6-4 photoproduct (**Figure 3** **lanes 25 to 33**). To date, no replicative DNA polymerase is able to bypass those UV-generated lesions and only specialized DNA polymerases are able to efficiently insert or elongate opposite these lesions [46], [47], [48].

### Fidelity of translesion DNA synthesis by EhDNApolB2

**Figure 4:**
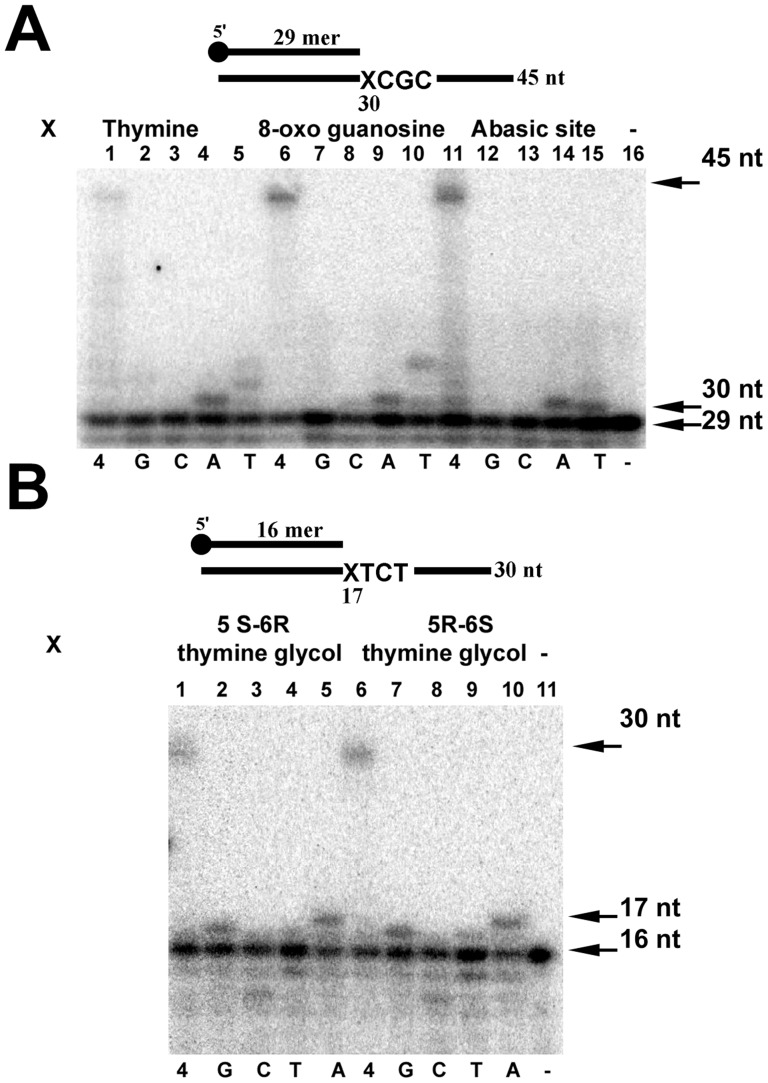
Fidelity of translesion DNA synthesis of EhDNApolB2. Translesion bypass fidelity of EhDNApolB 20 nM of exonuclease deficient EhDNApolB were incubated with 1 nM of a set of substrates containing several DNA lesions. The reactions were carried out with four dNTPs or single dNTP addition. The dNTPs were present at a concentration of 15 µM. Samples were taken at 2.5 minutes, stopped with 50 mM EDTA and 90% formamide and run onto a 18% denaturing polyacrylamide gel electrophoresis for their analysis by phosphorimagery. (A) Control thymine (lanes 1 to 5), 8-oxo guanosine (lanes 6 to 10), and abasic site (lanes 11 to 15). (B) 5 S-6R and 5R-6S thymine glycol (lanes 1 to 5 and 6 to 10 respectively). The upper arrow depicts the length of the final product substrate and the bottom arrow indicates the used primer.

EhDNApolB2 contains an active 3′–5′ exonuclease domain that in seconds degrades a labeled P-^32^ primer if a primer-templated duplex is annealed to an abasic site or thymine glycol in the absence of dNTPs at 37°C (data not shown). In order to test the fidelity of lesion bypass by EhDNApolB2 opposite to these lesions we constructed an exonuclease deficient polymerase Asp345Ala mutant that eliminates one of the essential carboxilates of motif ExoIII. We tested the fidelity of lesion bypass using as templates 8-oxo guanosine, abasic site, and thymine glycol. To avoid sequence context we used an undamaged 45mer template with identical sequence to the template containing 8-oxo guanosine and abasic site (**Table S4**). Using single dNTPs in independent reaction mixtures we observed that EhDNApolB2 incorporates dATP opposite a template thymine (**Figure 4A** **, lane 4**), misincorporates dTTP (**Figure 4A** **, lane 5**) and is unable to incorporate dGTP and dCTP opposite thymine (**Figure 4A** **, lanes 2 and 3**). EhDNApolB2 preferentially incorporates dATP opposite 8-oxo guanosine (**Figure 4A** **, lane** **9**) and misincorporates dTTP (**Figure 4A** **, lane 10**). As expected, EhDNApolB2 is unable to incorporate dGTP (**Figure 4** **, lane 7**), but unexpectedly EhDNApolB2 does not incorporate dCTP opposite 8-oxo guanosine (**Figure 4A** **, lane 8**). 8-oxo guanosine is a dual coding lesion in which the *syn* conformation of 8-oxo guanosine mimics a template thymine allowing dATP incorporation opposite the lesion [49], [50]. Several DNA polymerases like DNA polymerase I of *Bacillus stearothermophilus* incorporate dATP with high preference opposite from 8-oxo guanosine [50] by allowing the *syn* conformation of 8-oxo guanosine and DNA polymerase ι selectively incorporates dCTP opposite 8-oxo guanosine by presenting a narrow active site that does not allows the *syn* conformation of 8-oxo guanosine [51]. Thus, it is possible that EhDNApolB2 presents a specific interaction with 8-oxo guanosine that favors the *syn* over the *anti* conformation and thus favors dATP incorporation. DNA polymerases incorporate dATP or dGTP opposite an abasic site following the “A rule” [52]. As expect EhDNApolB2 preferentially incorporates dATP opposite an abasic site (**Figure 4A** **, lane 14**). The migration of the full-length primer extension product is in the same position in the abasic site, thymine and 8-oxoguanosine templates (**Figure 4A** **, lanes 1, 6 and 11**) indicating that EhDNApolB2 selectively incorporates dATP opposite and abasic site and that the mechanism of bypass does not involve skipping this lesion as is the case for DNA polymerase β [53]. This observation is corroborated by the migration of the 30mer band corresponding to dATP incorporation in thymine, 8-oxoguanosine and abasic site (**Figure 4A** **, lanes 4, 9, and 14**)

Exonuclease deficient EhDNApolB2 displays an increased bypass opposite thymine glycol isomers (**Figure 4B** **, lanes 1, 6 and 11**) in comparison to wild type (**Figure 4B** **, lanes 1 and 6**) as is observed for an exonuclease deficient RB69 DNA polymerase [45]. EhDNApolB2 preferentially incorporates dATP and dGTP opposite thymine glycol (**Figure 4B** **, lanes 2, 5, 7 and 10**). Although more detailed kinetic experiments are needed to understand lesion bypass fidelity of EhDNApolB2 the preliminary data indicates that EhDNApolB2 misincorporates thymine opposite a template thymine, abasic site, and 8-oxo guanosine and misincorporates dGTP opposite thymine glycol. This observation is consistent with that fact that family A polymerases involved in lesion bypass also incorporate with low fidelity [54], [55], [56].

### A mutant ΔTPR2 is active but with hampered polymerase and exonuclease activities

**Figure 5:**
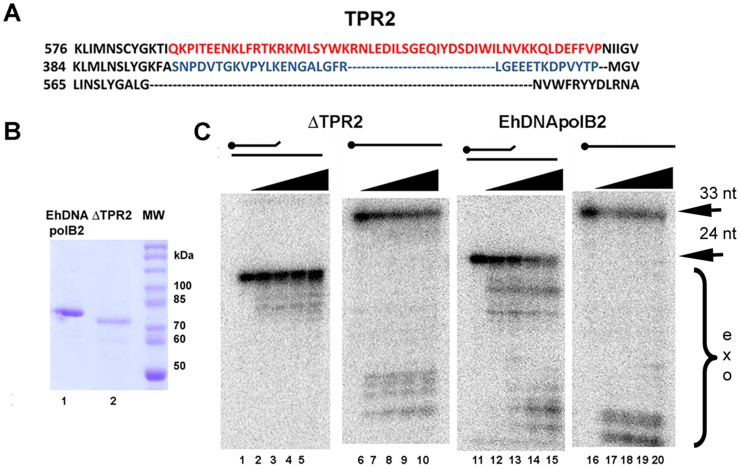
Role of extended TPR2 in exonuclease and polymerization activities. (**A**) Structural amino acid alignment of EhDNApolB2 in comparison to φ29 DNA polymerase and RB69 in the TPR2 region. TPR2 consists of 48 amino acids in EhDNApolB2, 24 amino acids in φ29 DNA polymerase and is absent in RB69. (**B**) Purification of ΔTPR2. 10% SDS-PAGE Coomassie blue stained showing the final purification of ΔTPR2 in comparison to EhDNApolB2. EhDNApolB2 is observed as a single protein band of 78 kDa (lane 1) in comparison to ΔTPR2 of 72 kDa (lane 2). (**C**) Autoradiogram showing the reaction products over the time course of 0, 2.5, 5, 10 and 20 min reaction by EhDNApolB2 and ΔTPR2 in the absence of dNTPs for a mismatched primer template (lanes 1 to 5 and 11 to 15) and single stranded DNA (lanes 6 to 10 and 16 to 20). Reactions were carried out using a radiolabeled primer as indicated in material and methods. Exonucleolytic activities were measured at 37°C.

EhDNApolB2 contains a TPR2 motif 21 amino acid longer than the one present in φ29 DNA polymerase (**Figure 5A**). Mutagenesis experiments have corroborated that TPR2 is involved in processivity and strand displacement in φ29 DNA polymerase [14]. As the TPR2 motif of EhDNApolB2 is twice the size of the orthologous φ29 DNA polymerase we hypothesized that this domain may have the same or novel functions. To determine the putative involvement of TPR2 in EhDNApolB2 lesion bypass we constructed a deletion mutant that eliminates 43 amino acids of TPR2 from EhDNApolB2. This mutants is readily purified using the same purification than wild-type EhDNApolB2 (**Figure 5B** **, lanes 1 and 2**). We measured the polymerization and exonuclease activities for EhDNApolB2 and ΔTPR2 using a fixed concentration of both polymerase in a time course reaction with and with out added dNTPs (**Figure 4S**). As observed wild-type EhDNApolB2 is able to efficiently elongate a primer template in a time dependent manner, however exonucleolytic degradation bands are also observed and accumulated over time (**Figure 4S** **, lanes 2 to 5**). In the other hand, the ΔTPR2 mutant is halted after the incorporation of three nucleotides suggesting a processivity problem at this position, perhaps triggered by a favored misinsertion (**Figure 4S** **, lanes 6 to 9**).

The 3′–5′ exonuclease activity of the ΔTPR2 mutant in the presence of nucleotides is drastically reduced (**Figure 4S** **, lanes 6 to 9**) suggesting that the extended TPR2 insertion has a role in the conformational changes that occurs during editing and polymerization modes. In order to discern the impact of ΔTPR2 during exonucleolytic degradation, the same experiment but without added dNTPs was performed. As shown before EhDNApolB2 contains a strong exonuclease activity and is able to efficiently degrade a 24mer to a 4mer in 20 minutes. On the other hand ΔTPR2 only degrades a 24mer to a 15mer after 20 minutes. (**Figure 4S** **, lanes 1 to 9**).

To further evaluate the role of TPR2 in proofreading and 3′–5′ exonuclease activity we performed a time course experiment using a mispaired primer-template and a single stranded labeled oligonucelotide (**Figure 5C**). During an incubation period from 2 to 10 minutes only 30% of the mispaired 24mer is degraded to products of 22 to 19 nucleotides by ΔTPR2 (**Figure 5C** **, lanes 1 to 5**). This is in contrast to the almost complete exonucleolytic 24mer degradation to products from 22 to 4 nucleotides by wild type EhDNApolB2 (**Figure 5C** **, lanes 11 to 15**). Suggesting a putative role of EhDNApolB2's TPR2 in exonucleolytic degradation of mispaired primers. φ29DNApolymerase's ΔTPR2 also presents a decay in exonucleolytic degradation in comparison to wild-type enzyme, however this domain is not involved in coordinating exonuclease and polymerization activities [14], [57]. In contrast mutation in intrinsic processive elements like the thioredoxin binding loop of T7 DNA polymerase diminish the extend of exonuclease activity indicating that other polymerases processive elements couple exonuclease and polymerization activities [58]. ΔTPR2 and wild-type EhDNApolB2 present a similar extended of exonucleolytic degradation at a single stranded DNA oligonucleotide. At the longest incubation time (10 minutes) approximately 60% of the substrate has been degraded. Interestingly ΔTPR2 degrades to 4–8mers whereas wild-type EhDNApolB2 degrades to 3–4mers (**Figure 5C** **, lanes 6 to 10 and 16 to 20**).

### Processive DNA polymerization by EhDNApolB2 depends on TPR2

**Figure 6:**
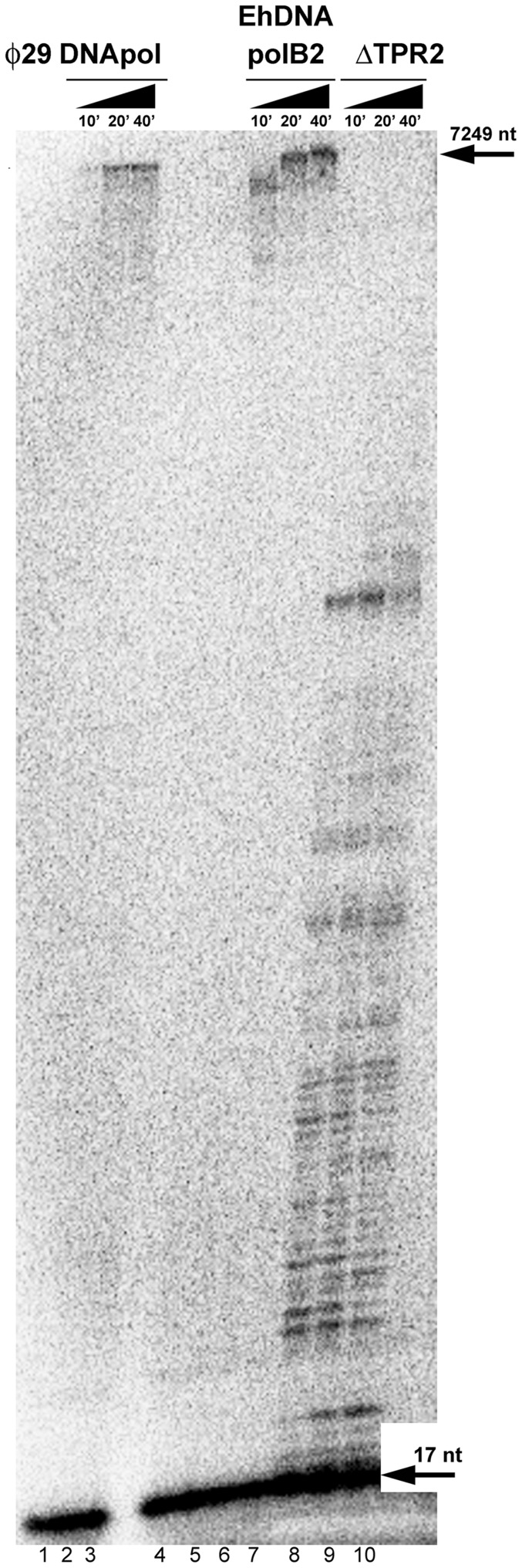
Processivity of EhDNApolB2 in comparison to φ29 DNA polymerase, and its dependence on TPR2. The processivity of wild-type EhDNApolB2 was measured in comparison to φ29 DNA polymerase and ΔTPR2. Reactions were carried out with 20 nM of the indicated polymerase and 1 nM of γ–P^32^17mer primer annealed to circular M13mp18 ssDNA. Aliquots were taken at 10, 20 and 40 min and loaded onto a 6% denaturing polyacrylamide gel. The arrows in the right correspond to the full-length M13 DNA amplification and abortive products.

Family B DNA polymerases interact with PCNA to increase their processivity. DNA polymerases α and δ from *E. histolytica* contain canonical PCNA binding motifs and are expected to be highly processive, as PCNA from *E. histolytica* (EhPCNA) assembles as a trimeric toroid [59]. EhDNApolB2 does not contain a canonical PIP box binding motif and full-length EhDNApolB2 is not stimulated by EhPCNA (data not shown). φ29 DNA polymerase is able to incorporate more than 70 kb of DNA in a single DNA binding event [60] and mutagenesis studies have demonstrated that the TPR2 motif of φDNA polymerase is involved in processivity [14]. To asses the intrinsic processivity of EhDNApolB2 we used a single stranded M13mp18 substrate annealed to a 17mer with equimolar amounts of control φ29 DNA polymerase and ΔTPR2 (**Figure 6**). After 40 minutes, full length M13mp18 is synthesized by φ29 DNA and EhDNApolB2 polymerases and no abortive/distributive products are observed (**Figure 6** **, lanes 1 to 3 and 5 to 7**) in comparison to a control primer template without added polymerase (**Figure 6** **, lane 4)**. As observed after 10 minutes, EhDNApolB2 completely extends a M13mp18 substrate and after a period of 20 to 40 minutes is able to perform a second round of synthesis over the same substrate displacing the newly synthesized DNA. Thus, EhDNApolB2 is more processive than φ29 DNA polymerase (**Figure 6** **, lanes 1 to 3**). As expected ΔTPR2 synthesized DNA in a distributive/abortive fashion, demonstrating that the TPR2 motif is crucial for processivity in this DNA polymerase (**Figure 6** **, lanes 8 to 10**).

### TPR2 is involved in strand displacement

**Figure 7:**
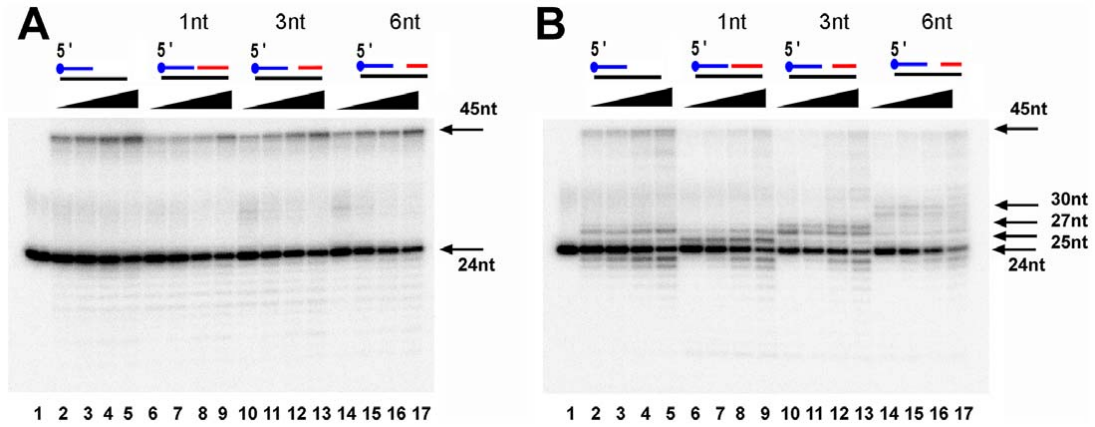
TPR2 is required for efficient strand-displacement. Strand displacement was assessed using a set of 3 oligonucleotides with gaps of 1, 3 and 6 nt respectively. After incubation at the indicated times the reaction mixtures were run on a 18% denaturing polyacrylamide gel. Reactions were carried out in 20 µl as described in material in methods (**A**) **Strand-displacement activity of EhDNApolB2**. Primer extension (lanes 2 to 5), primer extension with 1 nt gap (lanes 6 to 9), primer extension with 3 nt gap (lanes 10 to 13), primer extension with 6 nt gap (14 to 17). (**B**) **Strand-displacement activity of** Δ**TPR2** Primer extension (lanes 2 to 5), primer extension with 1 nt gap (lanes 6 to 9), primer extension with 3 nt gap (lanes 10 to 13), primer extension with 6 nt gap (14 to 17).

To corroborate the potentially strong strand displacement of EhDNApolB2 we prepared a set of four primer-template constructs in which a DNA polymerase should be able to fill a gap of 1, 3 and 6 nucleotides before displacing a duplex DNA and a control primer-template in which no strand-displacement is needed. After an incubation of 20 minutes, EhDNApolB2 efficiently displaces duplex DNA with gaps of 1, 3 and 6 nts with an efficiency of 21%, 27% and 34% in comparison to the control with out a 3′ annealed oligonucleotide that is extended with an efficiency of 32% (**Figure 7A** **, lanes 1 to 17**). In contrast the ΔTPR2 mutant only synthesizes full-length 45mer when a 3′ duplex barrier is not present (**Figure 7B** **, lanes 1 to 5**). In the presence of 1 nt gap, the ΔTPR2 mutant is halted at 25 nts and 27 nts and only 5% of the substrate is completely extended to the 45mer product. If the gap is of 3 nts, the mutant is halted at 27 nt and 28 nt and only 4% of the substrate is extended and if the gap is of 6 nts the polymerase is halted at 30 nt and only 2% of the substrate is fully extended (**Figure 7B** **, lanes 6 to 17**).

### ΔTPR2 confers lesion bypass opposite an abasic site

**Figure 8:**
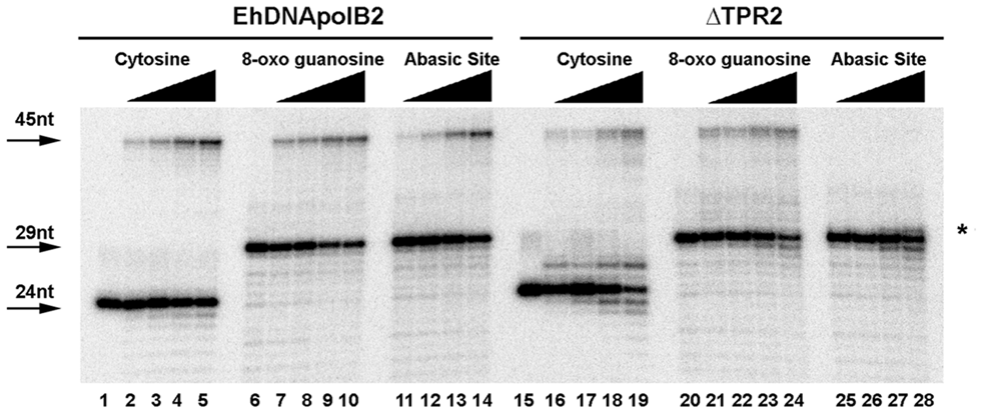
ΔTPR2 bypasses 8oxoG, but not an abasic site. Lesion bypass of EhDNApolB2 (lanes 1 to 14) and ΔTPR2 (lanes 15 to 28). The time course primer extension is described as in material and methods using equal amounts of DNA polymerases and 100 µM dNTPs. After incubation times of 2.5, 5, 10 and 20 minutes the primer extension reactions were stopped and run onto a 15% denaturing polyacrylamide gel.

Two structural solutions exists to improve the efficiency of DNA polymerases involved in translesion DNA synthesis: one solution is the presence of a wide active site in which a bulky lesion like a thymidine dimer can be easily accommodated, the other solution is the presence of extra insertions, like insertion 2 of DNA polymerase θ with respect to other family A DNA polymerases [61], [62]. In order to elucidate if the extra-length of TPR2 may have a role in lesion bypass, we carried out a time course DNA lesion bypass opposite an undamaged template, 8-oxoguanosine and an abasic site was assayed side by side with EhDNApolB2 and ΔTPR2. As previously demonstrated, EhDNApolB2 efficiently bypasses 8-oxoguanosine and abasic site (**Figure 8** **, lanes 1 to 14**). Interestingly the ΔTPR2 mutant efficiently bypasses 8-oxo guanosine but only incorporates 1 nt opposite an abasic site (**Figure 8** **, lanes 15 to 28**). In this experiment, the primer extension efficiencies having as a template cytosine, 8-oxo guanosine and abasic are 28%, 33% and 27% after 20 minutes of incubation (**Figure 8** **, lanes 1 to 14**). This is similar to extension efficiencies of ΔTPR2 in which 21% and 28% of the primer-template extension is observed using cytosine and 8-oxoguanosine, but in clear contrast to the extension opposite an abasic site, in which no fully 45mer product is observed. In this case 22% of the substrate is elongated only one nucleotide indicated by an asterisk.

**Figure 9:**
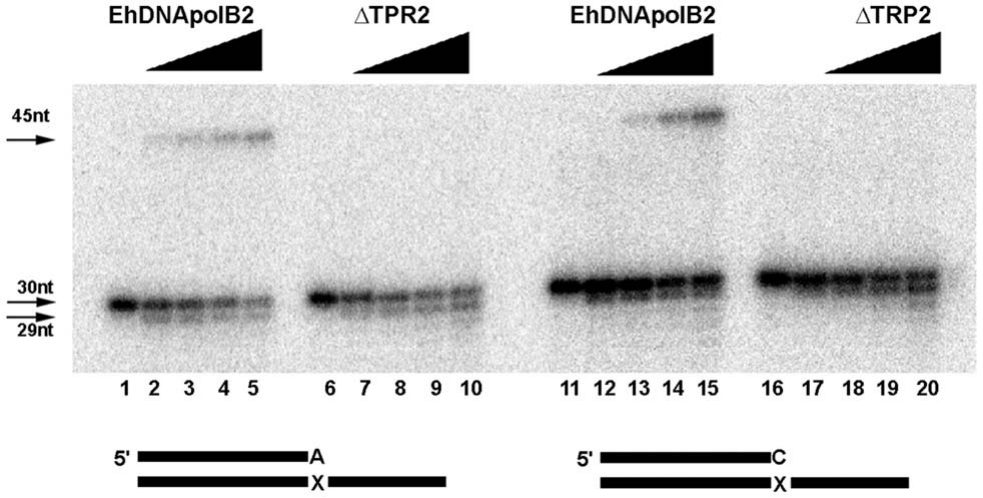
TPR2 is responsible of lesion bypass extension opposite an abasic site. Lesion bypass of wtEhDNApolB2 (lanes 1 to 5 and 11 to 15) and ΔTPR2 (lanes 6 to 10 and 16 to 20) extending from a primer containing a 3′OH purine or a pyrimidine opposite an abasic site. A primer containing a 3′OH dAMP (lanes 1 to 10) or dCMP (11 to 20) opposite an abasic site was subject to a time course primer extension reaction from 2.5 to 20 minutes using equal amounts of DNA polymerases and 100 µM dNTPs. The reaction products were run onto a 15% denaturing polyacrylamide gel.

EhDNApolB2 efficiently extends from a primer in which an oligonucleotide containing a 3′OH AMP or CMP overlap with an abasic site (**Figure 9** **, lanes 1 to 5 and 11 to 15**). In contrast ΔTPR2 is not able to extend this template that mimics the situation in which a nucleotide is incorporated opposite an abasic site. (**Figure 9** **, lanes 6 to 10 and 16 to 20**).

Thus, as in other DNA polymerases, EhDNApolB2 incorporates opposite and abasic site and TPR2 is the key element to pass this lesion. A recent report indicates that PCNA confers lesion bypass capabilities to DNA polymerase δ opposite an abasic site indicating an intrinsic ability of family B DNA polymerases to bypass this lesion [63]. The presence of the extra 21 amino acids in the TPR2 insert opens the possibility to speculate if this extra amino acids distort the active site to allow that an abasic site can be efficiently used as a non instructive template or if this TPR2 insertion contributes to an increased binding affinity of EhDNApolB2 that permits the polymerase extension from an abasic site.

## Supporting Information

Figure S1 **Phylogenetic analysis and Modular organization family B2 DNA polymerases.** (**A**) Phylogenetic analysis of the four family B2 DNA polymerases present in *E*. *histolytica* in relation to family B2 DNA polymerase from other protozoa, bacteriophages, and other eukaryotes. Accession numbers are indicated in Table S1. (**B**) **Modular organization of family B2 DNA polymerases in** ***E. histolytica*** **.** Modular organization of EhDNApolB2 (loci EHI_018010) in comparison to RB69 and Δ29 DNA polymerase. These family B2 DNA polymerases are composed of a 3′–5′ exonuclease domain and a 5′–3′ polymerization domain, with conserved motifs in both domains. EhDNApolB2 contains two Terminal Protein Region insertions dubbed TPR1 and TPR2 found in family B2 DNA polymerases as φ29 DNA polymerase [23], [27].
(TIF)

Figure S2 **Amino acid sequence alignment of RB69,** φ**29 DNA polymerase and EhDNApolB2.** Amino acid sequences were aligned using ClustalW. The conserved motifs in the exonuclease domains are indicated as ExoI, ExoII and ExoIII whereas the conserved motifs in the polymerase domain are indicated as A, B, and C. The YxGG/A motif involved in terminal protein interaction and the KXY motif involved in stabilizing the primer terminus [23], [24], [25], [27]. The consensus sequences for each motif are in bold. The extended TPR2 is colored in blue.
(TIF)

Figure S3 **Inhibition of EhDNApolB2 by aphidicolin.** Percentage of DNA elongation activity of EhDNApolB2 using a γ-P^32^ 17mer primer annealead to a circular ssDNA M13mp18 substrate in the presence of increasing aphidicolin concentrations. Reactions contained 20 nM of purified EhDNApolB2, 1 nM of circular substrate and increasing concentration of aphidicolin (0 to 640 µM). Reactions were incubated for 10 min to 37°C and loaded onto a 6% denaturing polyacrylamide gel. The inset shows the final elongation product. Primer elongation reactions were carried out by duplicate.
(TIF)

Figure S4 **Exonuclease and polymerization activities of EhDNApolB2 and ΔTPR2.** Reactions for panels A and B were carried out using a radiolabeled primer annealed to a complementary template as indicated in material and methods for EhDNApolB2 and **Δ**TPR2 in the presence (A) and absence (B) of dNTPs. (**A**)Autoradiogram showing the reaction products over a time course of 2.5, 5, 10 and 20 minutes by EhDNApolB2 and **Δ**TPR2 in the presence of dNTPs. (**B**) Autoradiogram showing the reaction products over a time course of 2.5, 5, 10 and 20 minutes by EhDNApolB2 and **Δ**TPR2 in the absence of dNTPs. Polymerization and exonucleolytic products are indicated by arrows. Polymerization an exonucleolytic activities were measured using a molar excess of EhDNApolB2 or ΔTPR2 to assure that the concentrations of active polymerases is greater than the substrate concentration.
(TIF)

Table S1 ***Entamoeba histolytica*** **family B2 DNA polymerases.**
(DOC)

Table S2 **Genbank identifiers of family B2 DNA polymerases.**
(DOC)

Table S3 **Oligonucleotides used for cloning and mutagenesis.**
(DOC)

Table S4 **Oligonucleotides used in primer extension and exonuclease reactions.**
(DOC)
