# Supplementary figures and images for: A Transposon-Derived DNA Polymerase from Entamoeba histolytica Displays Intrinsic Strand Displacement, Processivity and Lesion Bypass
Source: PLoS One. 2012 Nov 30;7(11):e49964. doi: 10.1371/journal.pone.0049964 (PMC3511435; doi:10.1371/journal.pone.0049964)

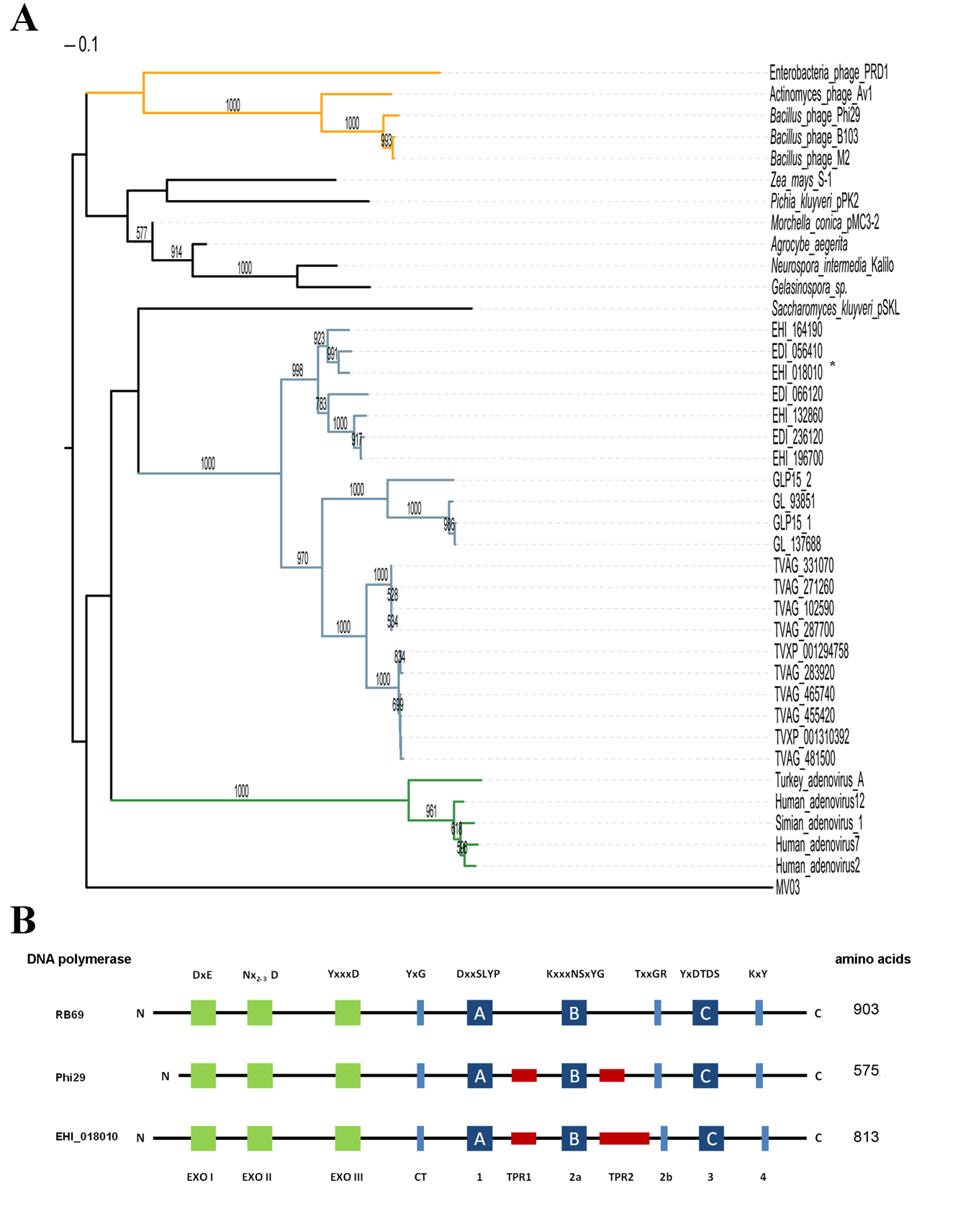

Supplement: Figure S1 — Phylogenetic analysis and Modular organization family B2 DNA polymerases. (A) Phylogenetic analysis of the four family B2 DNA polymerases present in E. histolytica in relation to family B2 DNA polymerase from other protozoa, bacteriophages, and other eukaryotes. Accession numbers are indicated in Table S1. (B) Modular organization of family B2 DNA polymerases in E. histolytica . Modular organization of EhDNApolB2 (loci EHI_018010) in comparison to RB69 and Δ29 DNA polymerase. These family B2 DNA polymerases are composed of a 3′–5′ exonuclease domain and a 5′–3′ polymerization domain, with conserved motifs in both domains. EhDNApolB2 contains two Terminal Protein Region insertions dubbed TPR1 and TPR2 found in family B2 DNA polymerases as φ29 DNA polymerase [23], [27]. (TIF) [file pone.0049964.s001.tif]

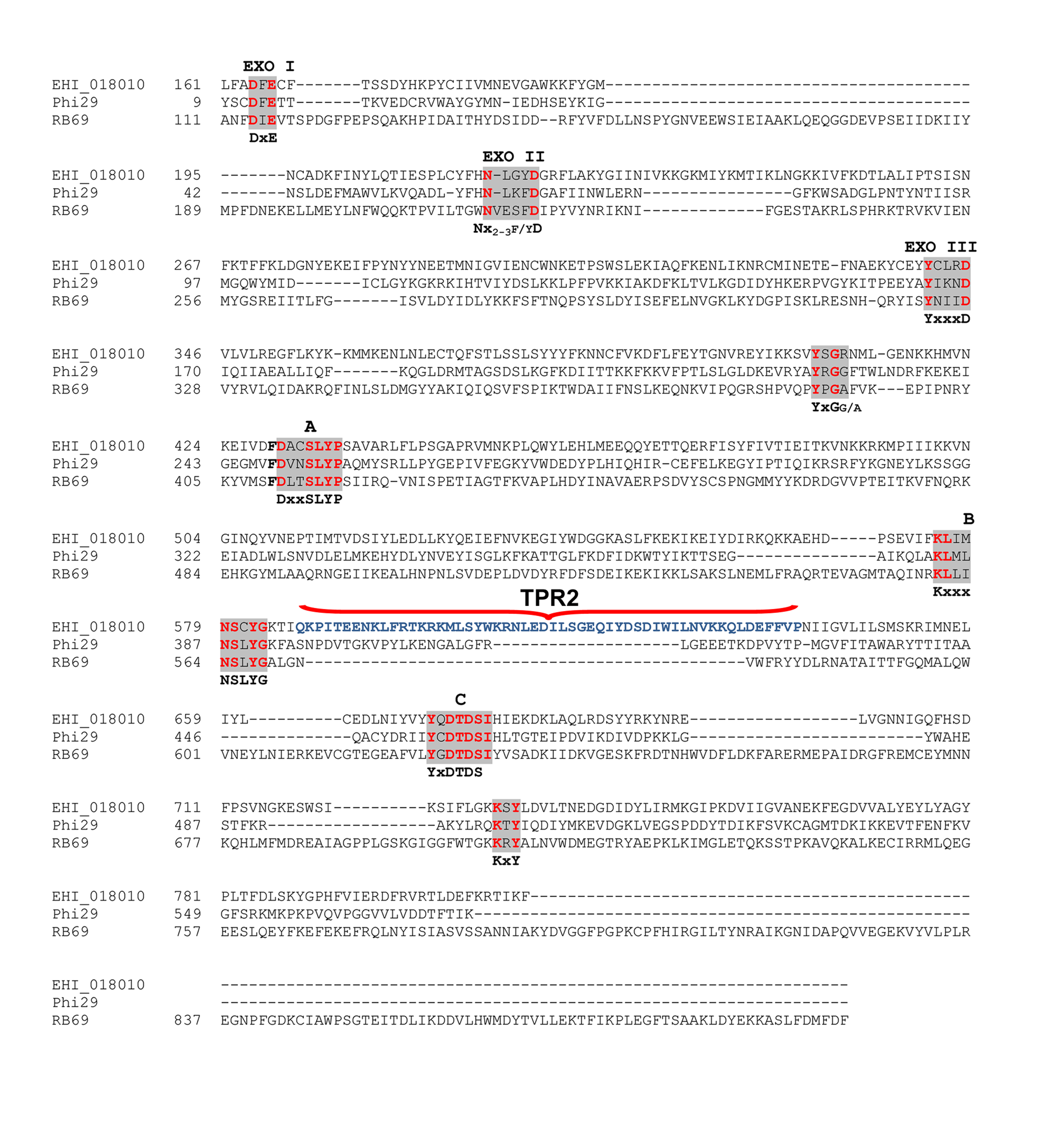

Supplement: Figure S2 — Amino acid sequence alignment of RB69, φ29 DNA polymerase and EhDNApolB2. Amino acid sequences were aligned using ClustalW. The conserved motifs in the exonuclease domains are indicated as ExoI, ExoII and ExoIII whereas the conserved motifs in the polymerase domain are indicated as A, B, and C. The YxGG/A motif involved in terminal protein interaction and the KXY motif involved in stabilizing the primer terminus [23], [24], [25], [27]. The consensus sequences for each motif are in bold. The extended TPR2 is colored in blue. (TIF) [file pone.0049964.s002.tif]

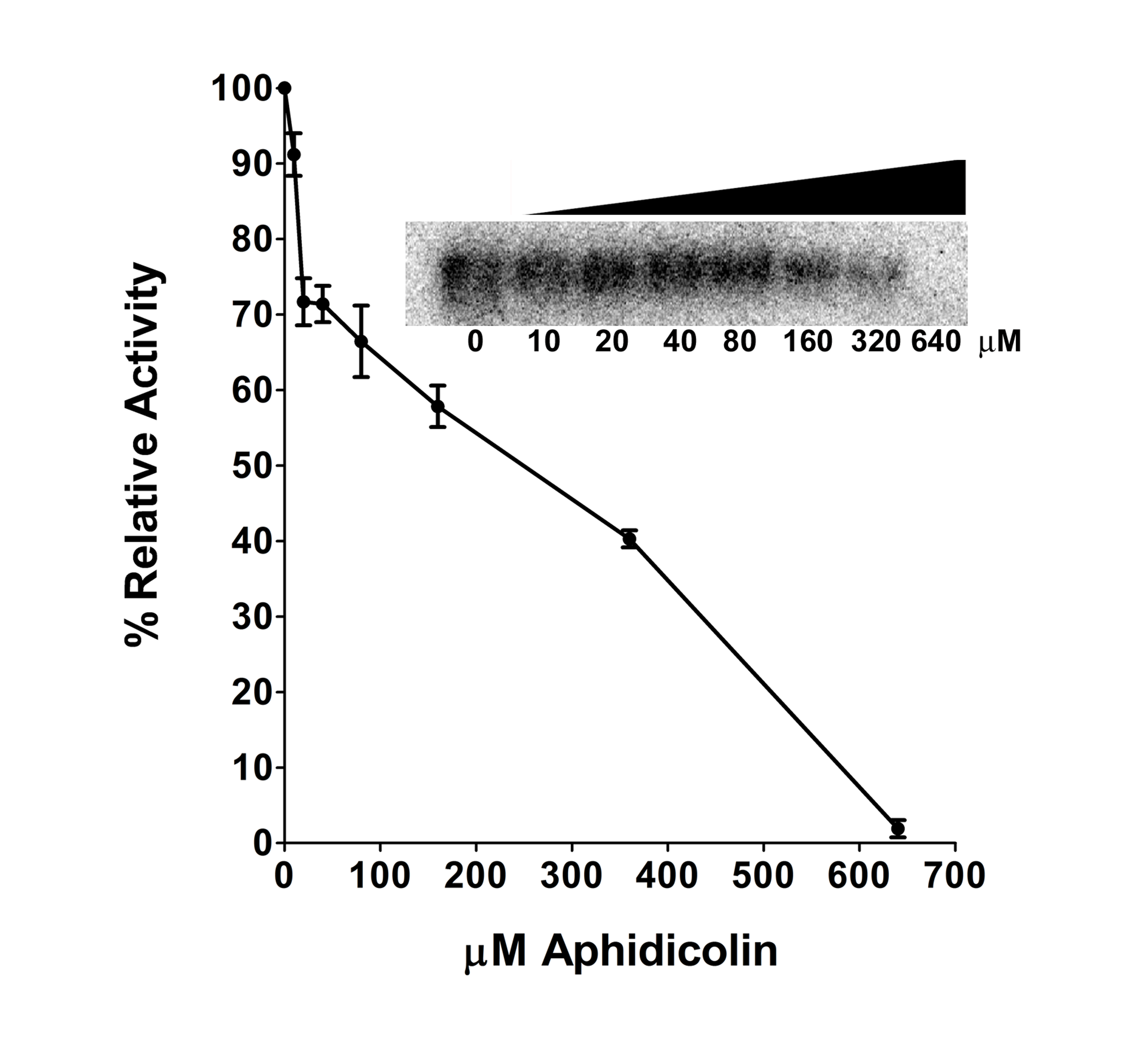

Supplement: Figure S3 — Inhibition of EhDNApolB2 by aphidicolin. Percentage of DNA elongation activity of EhDNApolB2 using a γ-P32 17mer primer annealead to a circular ssDNA M13mp18 substrate in the presence of increasing aphidicolin concentrations. Reactions contained 20 nM of purified EhDNApolB2, 1 nM of circular substrate and increasing concentration of aphidicolin (0 to 640 µM). Reactions were incubated for 10 min to 37°C and loaded onto a 6% denaturing polyacrylamide gel. The inset shows the final elongation product. Primer elongation reactions were carried out by duplicate. (TIF) [file pone.0049964.s003.tif]

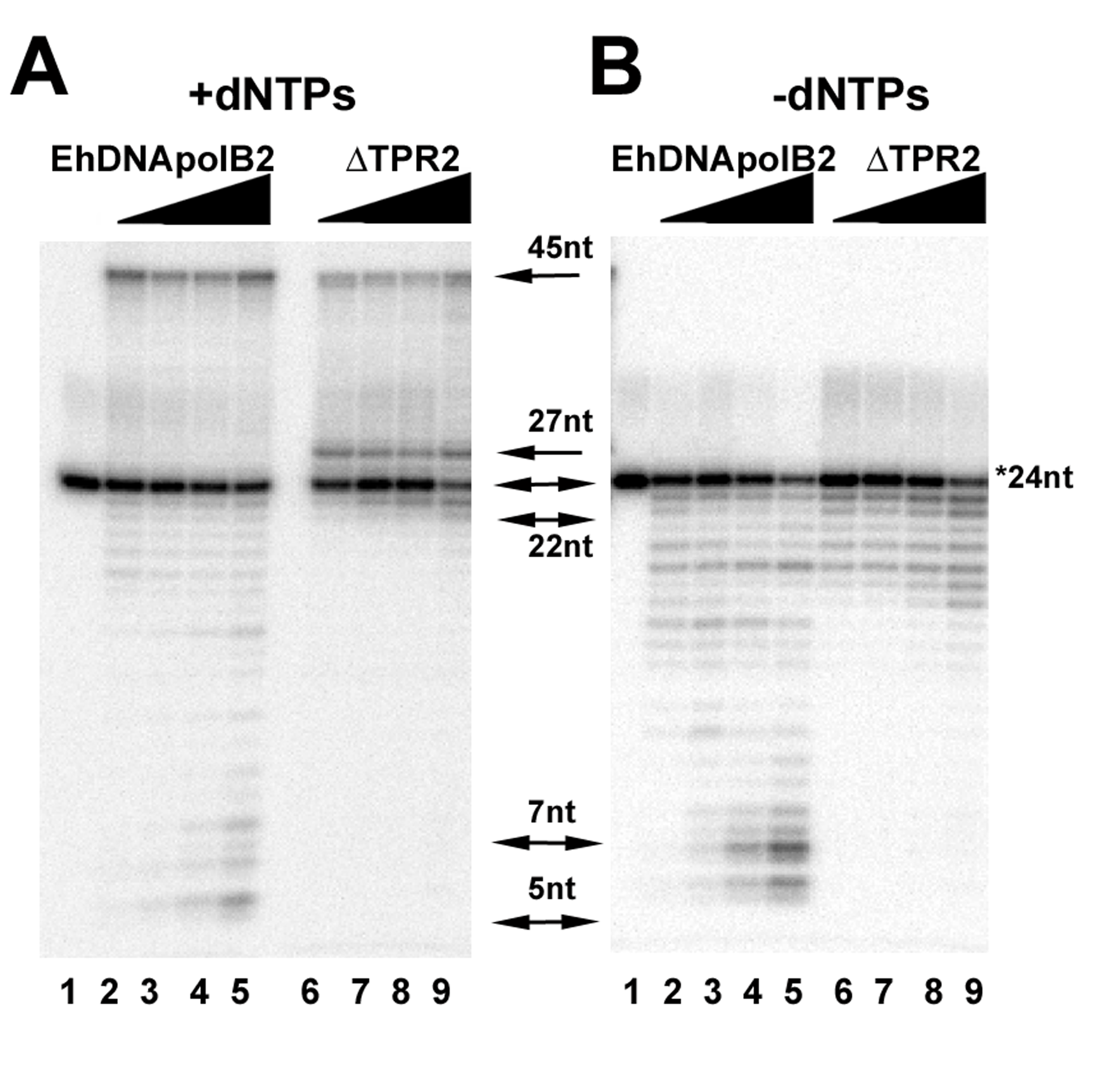

Supplement: Figure S4 — Exonuclease and polymerization activities of EhDNApolB2 and ΔTPR2. Reactions for panels A and B were carried out using a radiolabeled primer annealed to a complementary template as indicated in material and methods for EhDNApolB2 and ΔTPR2 in the presence (A) and absence (B) of dNTPs. (A)Autoradiogram showing the reaction products over a time course of 2.5, 5, 10 and 20 minutes by EhDNApolB2 and ΔTPR2 in the presence of dNTPs. (B) Autoradiogram showing the reaction products over a time course of 2.5, 5, 10 and 20 minutes by EhDNApolB2 and ΔTPR2 in the absence of dNTPs. Polymerization and exonucleolytic products are indicated by arrows. Polymerization an exonucleolytic activities were measured using a molar excess of EhDNApolB2 or ΔTPR2 to assure that the concentrations of active polymerases is greater than the substrate concentration. (TIF) [file pone.0049964.s004.tif]
