# Supplementary material for: A Transposon-Derived DNA Polymerase from Entamoeba histolytica Displays Intrinsic Strand Displacement, Processivity and Lesion Bypass
Source: PLoS One. 2012 Nov 30;7(11):e49964. doi: 10.1371/journal.pone.0049964 (PMC3511435; doi:10.1371/journal.pone.0049964)
Supplement: Table S1 — Entamoeba histolytica family B2 DNA polymerases. (DOC) [file pone.0049964.s005.doc]

**Table S1.** *Entamoeba histolytica* family B2 DNA polymerases

| *Loci* | Total amino acid length | Length  N-terminal | Length  C-terminal | % identity  C-terminal |
| --- | --- | --- | --- | --- |
| EHI_018010 | 813 | 153 | 660 | -------- |
| EHI_164190 | 1279 | 618 | 661 | 72 |
| EHI_196700 | 1231 | 569 | 662 | 61 |
| EHI_132860 | 1090 | 428 | 662 | 61 |
