# Supplementary material for: A Transposon-Derived DNA Polymerase from Entamoeba histolytica Displays Intrinsic Strand Displacement, Processivity and Lesion Bypass
Source: PLoS One. 2012 Nov 30;7(11):e49964. doi: 10.1371/journal.pone.0049964 (PMC3511435; doi:10.1371/journal.pone.0049964)
Supplement: Table S2 — Genbank identifiers of family B2 DNA polymerases. (DOC) [file pone.0049964.s006.doc]

**Table S2.** Genbank identifiers of family B2 DNA polymerases

| **Organism** | **Genbank** |
| --- | --- |
| Bacillus phage φ29 | YP_002004529.1 |
| Bacillus phage B103 | NP_690635.1 |
| Bacillus phage M2 | P19894.1 |
| Enterobacteria phage | AAX45903.1 |
| Zea mays | P10582 |
| *Neurospora intermedia* | NP_053000.1 |
| *Gelasinospora sp* G114 Gel-Kal | AAB41447.1 |
| *Morchella conica* | CAA45364.2 |
| *Actinomyces phage Av1* | YP_001333659.1 |
| *Saccharomyces kluyveri* | CAA38621.1 |
| *Pichia kluyveri pPK2* | CAA72340.1 |
| *Agrocybe aegerita* | AAC33727.1 |
| Simian adenovirus | YP_213966.1 |
| Human adenovirus | NP_040853.1 |
| Human adenovirus | AAT97530.2 |
| Human adenovirus | AP_000166.1 |
| Turkey adenovirus | AP_000478.1 |
| *Trichomonas vaginalis* | XP_001310451.1 |
| *Trichomonas vaginalis* | XP_001322658.1 |
| *Trichomonas vaginalis* | XP_001296546 |
| *Trichomonas vaginalis* | XP_001321733.1 |
| *Trichomonas vaginalis* | XP_001317079.1 |
| *Trichomonas vaginalis* | XP_001300959 |
| *Trichomonas vaginalis* | XP_001298011.1 |
| *Trichomonas vaginalis* | XP_001294758 |
| *Trichomonas vaginalis* | XP_001310392 |
| *Entamoeba histolytica* | XP_001914292.1 |
| *Entamoeba histolytica* | XP_649845.2 |
| *Entamoeba histolytica* | XP_001913700.1 |
| *Entamoeba histolytica* | XP_648196.2 |
| *Entamoeba dispar* | XP_001740480.1 |
| *Entamoeba dispar* | XP_001738693.1 |
| *Entamoeba dispar* | XP_001741766.1 |
| *Giardia lamblia ATCC 50803* | XP_001707491. |
| *Giardia lamblia ATCC 50803* | XP_001707488.1 |
| *Giardia lamblia P15* | EFO61616.1 |
| *Giardia lamblia P15* | EFO61805.1 |
| Mavirus | YP_004300281 |
