# Supplementary material for: A Transposon-Derived DNA Polymerase from Entamoeba histolytica Displays Intrinsic Strand Displacement, Processivity and Lesion Bypass
Source: PLoS One. 2012 Nov 30;7(11):e49964. doi: 10.1371/journal.pone.0049964 (PMC3511435; doi:10.1371/journal.pone.0049964)
Supplement: Table S3 — Oligonucleotides used for cloning and mutagenesis. (DOC) [file pone.0049964.s007.doc]

**Table S3.** Oligonucleotides used for cloning and mutagenesis

| Oligonucleotides full-length |  |
| --- | --- |
| N-terminal | 5´ gggaaaggattcatgatacattttgcagaagaattta 3´ |
| C-terminal | 5' ggaaggaagcttttaaaatttaattgttcttttgaac 3´ |
| Oligonucleotides ΔN-terminal |  |
| ΔN-terminal | 5' gggaaaggattcagtgaaactactaatacattg 3´ |
| C-terminal | 5' ggaaggaagcttttaaaatttaattgttcttttgaac 3´ |
| Oligonucleotides ΔTPR2 |  |
| ΔTPR2-Fw | 5´ caattggacgaattcttcgttcctaata 3´ |
| ΔTPR2-Rev | 5´ tggcttctgaatagttttgccataacagg 3´ |
| PolExo_Asp345Ala Fw | 5´ gtgaatattattgtttaagggccgtgttagtgttaagagaagg3´ |
| PolExo_Asp345Ala Rev | 5´ ccttctcttaacactaacacggcccttaaacaataatattcac3´ |
