# Supplementary material for: A Transposon-Derived DNA Polymerase from Entamoeba histolytica Displays Intrinsic Strand Displacement, Processivity and Lesion Bypass
Source: PLoS One. 2012 Nov 30;7(11):e49964. doi: 10.1371/journal.pone.0049964 (PMC3511435; doi:10.1371/journal.pone.0049964)
Supplement: Table S4 — Oligonucleotides used in primer extension and exonuclease reactions. (DOC) [file pone.0049964.s008.doc]

**Table S4 Oligonucleotides used in primer extension and exonuclease reactions**

| **Canonical substrate** |  | |
| --- | --- | --- |
| Primer | 5´ cgcagcccacctgcccacctaact 3´ | |
| Template cytosine | 5´ ccttggcactagcgcagggccagttaggtgggcaggtgggctgcg 3´ | |
| Primer misparied | 5´ cgcagcccacctgcccacctaacc 3´ | |
| Primer Strand displacement |  | |
| 1 nt gap | 5´ gccctgcgctagtgccaagg 3´ | |
| 3 nt gap | 5´ cctgcgctagtgccaagg 3´ | |
| 6 nt gap | 5´ gcgctagtgccaagg 3´ | |
| **Lesion bypass** | | |
| Primer abasic site and 8-oxo guanosine | | 5´ cgcagcggacctgcccacctaactgatat 3´ |
| Template abasic site and 8-oxo guanosine | | 5´ccttggcactagcgcXatatcagttaggtgggcaggtccgctgcg 3´ |
| Template thymine | | 5´ccttggcactagcgctatatcagttaggtgggcaggtccgctgcg 3´ |
| Primer thymine glycol | | 5´ cactgactgtatgatg 3´ |
| Template thymine glycol, CPD and 6-4 photoproduct | | 5´ ctcgtcagcatctXcatcatacagtcagtg 3´ |
| Lesion bypass extension opposite an abasic site | | |
| Primer C | | 5´ cgcagcggacctgcccacctaactgatatc 3´ |
| Primer A | | 5´ cgcagcggacctgcccacctaactgatata 3´ |
| **Processivity** | |  |
| Primer M13mp18 | | 5´ gttttcccagtcacgac 3´ |
